# HEOCP: Hybrid Energy-Optimized Clustering Protocol for WSNs Using Analytical Modeling and Deep Learning Integration

**DOI:** 10.3390/s26041188

**Published:** 2026-02-12

**Authors:** Yen-Wu Ti, Rei-Heng Cheng, Songlin Wei, Chih-Min Yu

**Affiliations:** 1 School of Information Engineering, Xiamen Ocean Vocational College, Xiamen 361100, China; diyanwu@xmoc.edu.cn (Y.-W.T.); zhengruiheng@xmoc.edu.cn (R.-H.C.); 2 Fujian Provincial Key Laboratory of Marine Physical and Geological Processes, Xiamen 361005, China; 3 Department of Information and Computer Engineering, Chung Yuan Christian University, Taoyuan 320314, Taiwan; ycm@cycu.edu.tw

**Keywords:** wireless sensor networks, LEACH protocol, genetic algorithm

## Abstract

**Highlights:**

**What are the main findings?**

- A derivable and fully solvable energy-consumption model was developed, allowing us to determine both the optimal distance range and the ideal number of clusters for selecting Cluster Heads (CHs).
- Proposes a hybrid CH selection framework that leverages ResNet-50 to capture the spatial features of the wireless sensor network (WSN) and smooth out the results across multiple rounds of a genetic algorithm (GA). By doing so, it eliminates the need for expensive real-time computations.
- The proposed Hybrid Energy-Optimized Clustering Protocol significantly extends the network lifetime under various WSNs scales.

**What are the implications of the main findings?**

- An energy optimization theoretical framework is provided that can be widely applied to large-scale or dynamic WSNs.
- Using deep learning to decrease computational needs makes “intelligent CH selection” possible in real-world IoT systems.
- The Hybrid AI + Analytical Modeling approach has been shown to be beneficial in extending the lifetime of WSNs.

**Abstract:**

Wireless Sensor Networks (WSNs) play a pivotal role in Internet of Things (IoT) applications; however, their lifetime is fundamentally constrained by the limited energy of sensor nodes. This paper introduces a Hybrid Energy-Optimized Clustering Protocol (HEOCP) that combines analytical modeling of radio energy consumption with deep learning–assisted cluster-head (CH) selection. First, an analytical framework is developed to determine the distance-constrained CH eligibility region and the optimal number of clusters, thereby minimizing redundant transmissions and balancing energy consumption. Then, a genetic algorithm (GA) is used to determine the best cluster head configuration. These configurations are then trained by a ResNet-50 deep network and averaged to reduce noise, allowing for real-time cluster head prediction without repeatedly performing expensive heuristic optimization, resulting in more steady performance. Extensive simulations under various network scales demonstrate that HEOCP extends network lifetime by up to 60% compared with conventional LEACH and GA-based approaches, effectively delaying the first-node death and improving overall energy efficiency. Furthermore, the hybrid GA–ResNet framework exhibits high scalability and computational efficiency, making it suitable for large-scale IoT deployments. The results confirm that integrating analytical energy modeling with deep learning provides a powerful and sustainable paradigm for intelligent energy management in future IoT-enabled WSNs.

## 1. Introduction

In recent years, the rapid development of semiconductor technology, battery technology, and wireless communication technology has enabled small sensors to perform wireless communication transmission and data aggregation processing [1], leading to a growing number of application scenarios for wireless sensor nodes. This trend has accelerated the development of wireless sensor networks (WSNs), which are now an important area of research. WSNs are widely used in a variety of applications, including environmental and ecological monitoring, health monitoring, and home automation [2]. A key challenge in WSN design is to reduce sensor energy consumption and increase network operating time [3]. Since WSNs are often deployed in hard-to-reach locations, many of their sensors rely on batteries; however, it can be prohibitively expensive or even infeasible to replace or recharge batteries in these areas. When sensor batteries are depleted, parts of the network may fail, potentially leading to the paralysis of the entire network. Therefore, how to effectively reduce the energy consumption of each sensor in the network and delay the time when the sensor runs out of energy to prolong the network lifetime has become the current research focus on WSNs [4].

A major factor in evaluating the efficacy of a WSN is the lifetime of its sensors and the network as a whole [5]. Although every sensor in a WSN has the capability to send data to the sink, not all sensors are well-suited for direct communication with it due to distance constraints. In practical applications, direct communication between sensors and the sink is inefficient when distant nodes must expend excessive energy to transmit data. Therefore, selecting some sensors located closer to the sink to act as relay nodes for transmitting messages can be beneficial. Sensors farther from the sink send their messages to these relay nodes, which then collect the messages and transmit them to the sink. However, this approach increases the energy consumption of the sensors acting as relay nodes, potentially causing them to exhaust their energy quickly. Therefore, an attractive approach is to adopt a clustering architecture in which selected sensors act as relay nodes and are designated as Cluster Heads (CHs) [6]. In a clustered sensor network, a particular algorithm will select a sensor for the CH. This sensor is responsible for transmitting the data received by the sensors in its cluster to the sink node. However, CHs need to receive signals from other sensors in their cluster, thus consuming more energy and making their lifetime shorter than ordinary sensors. Thus, reducing the average energy consumption of CHs has become an important research topic [7]. Prior studies have shown that selecting appropriate CH locations and an appropriate number of CHs can reduce overall network energy consumption and extend network lifetime [8,9,10].

Conventional clustering protocols, such as low-energy adaptive clustering hierarchy (LEACH) and its derivatives, primarily rely on probabilistic CH selection schemes that ignore the spatial relationship between sensors and the sink [6]. In particular, traditional clustering approaches make two major deficiencies during the decision-making process. On the one hand, they neglect the impact of sensor distribution density on optimal cluster sizing; on the other hand, they fail to establish a quantitative relationship between the monitored area and energy-balancing objectives. These deficiencies often lead to inefficient clustering strategies and shorter network lifespans.

Motivated by these challenges, this work introduces the Hybrid Energy-Optimized Clustering Protocol (HEOCP), which integrates distance-aware clustering design with intelligent energy management. The key contributions of this paper are as follows:

1. We analytically establish a distance-constrained CH eligibility region using a model for radio energy consumption, thus creating a mathematical basis for finding the ideal relay nodes for signal transmission in WSN clustering strategies.
2. We provide a GA-to-ResNet surrogate (teacher-student) structure that turns the GA’s CH selections for each round into a single forward-pass predictor, which lets us get near the performance of the GA at a much lower runtime. A hybrid optimization framework based on the residual neural network 50 (ResNet-50) deep learning model is proposed, which learns optimization results from multiple rounds of a genetic algorithm (GA) to form a smooth and stable decision model. This framework enables dynamic CH selection by jointly optimizing energy consumption and historical patterns of sensor utilization.
3. We developed a cluster scale estimate approach to avoid selecting too few CHs, which could make the sensors that are selected as CHs use too much energy. This model is also used to make the ResNet-50 model better at selecting CHs.

Together, these contributions significantly improve the energy efficiency and spatial adaptability of WSNs and provide useful tools for operators to reduce deployment and maintenance costs.

The remainder of this article is organized as follows: Section 2 introduces the relevant research results. Section 3 derives the appropriate CH selection area and the optimal number of clusters in the clustering mechanism based on the wireless sensor network model. Section 4 then introduces our proposed HEOCP clustering protocol. Section 5 presents our experimental results and includes comparisons with other research results. Section 6 summarizes our results and provides conclusions.

## 2. Related Works

To overcome the shortcomings of the random CH selection in LEACH, numerous studies have introduced advanced optimization algorithms and decision models to elect more energy-efficient CHs.

Regarding the application of GA and swarm intelligence, Rani et al. proposed a dynamic clustering method based on GA combined with Relay Nodes (RN) to optimize data transmission [11]. Nandan et al. presented the Optimized GA Cluster Head Election (OptGACHE) algorithm [12]. Similarly, Nagarajan et al. combined the Jaya algorithm with SailfishOptimization to design a multi-objective LEACH protocol [13]. In addition, Wang et al. proposed ICGA-LEACH, which employs a chaotic genetic algorithm to jointly determine cluster members and multi-hop routing paths using a fitness function that considers both energy consumption minimization and load balancing, and further introduces an adaptive round time to reduce energy consumption and prolong network lifetime [14].

Beyond WSN-oriented LEACH improvements, topology-aware evolutionary strategies have also been explored. For example, Huang et al. combined complex-network modeling (Barabási–Albert scale-free structure) with genetic algorithms and machine learning to enhance the search process [15].

Beyond the above, many other optimization strategies have been applied to improve the clustering mechanism in WSNs. Gülbaş and Çetin employed the Simulated Annealing (SA) algorithm to optimize the CH selection process, minimizing node energy loss [16]. Gangal et al. integrated the Analytic Hierarchy Process (AHP) with the LEACH protocol to form the LEACH-AHP routing scheme, which aids distributed clustering decisions [17]. Furthermore, Jurado-Lasso et al. explored the application of Reinforcement Learning (RL) to optimize clustering and IoT data transmission with the LEACH-RLC protocol [18]. Collectively, these methods demonstrate the capability of using complex algorithms to precisely control a CH election.

Moreover, by altering the network’s geographical structure, it is possible to effectively balance the load and reduce the energy consumption of long-distance transmissions. Korteby and Gál examined the effects of integrating a mobile sink within a heterogeneous network environment on the LEACH protocol, concluding that mobility enhances energy distribution uniformity [19]. Wei et al. further proposed the Dynamic Spanning Tree with Mobile Sink (DSTMS) routing algorithm to optimize data transmission paths in dynamic environments [20].

Regarding regional partitioning strategies in WSNs, Mohammed et al. proposed Sectored LEACH (S-LEACH), which divides the communication area into sectors to shorten transmission distances, thereby reducing overall energy consumption [21]. Sinde et al. combined angle sector partitioning with energy-aware TDMA scheduling to further enhance network lifetime [22].

The comprehensive consideration of multiple physical and network parameters during the CH election is also key to improving LEACH performance. For multi-factor decision applications, Huang et al. proposed the NF-LEACH algorithm, which considers factors such as residual energy, distance to the base station (BS), and data transmission mode to improve CH selection [23]. Salman et al.’s work primarily focused on optimizing CH election and cluster formation based on the sensor’s current energy and distance to the base station [24].

Furthermore, in research on WSN clustering and energy consumption balance, Chang et al. addressed the non-uniform cluster partitioning problem in the LEACH protocol by proposing an improved clustering algorithm based on node distribution density to achieve rapid cluster division and energy balance [25]. The NM-LEACH protocol by Abdulaal et al. also focused on the problem of uneven energy consumption caused by not considering the distance between the cluster head and member nodes [26].

For WSN stability and specific optimizations, Al-Zubaidi et al. re-evaluated Stable Improved LEACH (SILEACH), which aims to increase the network’s stability period—a crucial factor for applications requiring reliable feedback [27]. Rajaram et al. proposed the Enriched Energy Optimized LEACH (EE-OLEACH) protocol, designed to achieve efficient data transmission through optimized clustering and routing selection mechanisms [28]. In addition to purely energy-efficient clustering/routing, some studies pursue “energy sustainability” by integrating energy replenishment mechanisms. Lin et al. [29] proposed ESWCM for SWIPT-enabled WSNs under constrained Mobile Energy Access Point (MEAP) configurations, including optimal ring-width determination (ORWD), a GA-based clustering method constrained by cluster-head connectivity (GCH), and per-node energy-efficient parameter optimization (EPO).

The following works concentrate on prolonging the overall network lifespan via comprehensive protocols or integrated enhancements. Nasr and Quwaider put forward a new way to improve the LEACH protocol, with the goal of extending the life of the network and the time it takes to send data [30]. Daanoune and Baghdad focused on improving the BRE-LEACH protocol, proposing IBRE-LEACH to address the issue of “Abandoned Nodes” in the network, a specific but important energy consumption problem [31]. Pankaj and Sharma’s research showed that adding a better version of the LEACH protocol to a network can help it last longer [32].

Despite the significant improvements made to the LEACH protocol in existing literature across single aspects (e.g., meta-heuristic optimization, mobility, multi-parameter decision-making), several challenges remain. Firstly, while many AI/metaheuristic-based methods achieve satisfactory optimization results, their high computational complexity makes them difficult to execute efficiently on severely resource-constrained sensor nodes. Furthermore, existing solutions for addressing the uneven energy consumption of CHs (especially those near the base station) or non-uniform cluster distribution often involve only a single dimension.

This study presents a highly integrated, low-complexity multi-objective CH election mechanism. We propose an agent-based (teacher-student) framework that transfers knowledge from a GA to a ResNet model, distilling the GA’s per-round CH selection decisions into a single forward-pass predictor that achieves near-GA performance while requiring only a fraction of the runtime. To our knowledge, existing research on CH selection in WSNs has not explicitly formulated the CH selection problem as one where an image-based surrogate model learns the per-round decisions of a metaheuristic “teacher” algorithm during inference, thereby replacing the traditional iterative optimization process.

## 3. The Model of Wireless Sensor Networks

The core concept of the clustering algorithm proposed in this study shares similarities with well-established methods in the field, such as the LEACH protocol. This study adopts a standard WSN research configuration. The network consists of sensor nodes that have limited energy and a sink that receives all the messages. The sensors can communicate to each other, receive messages from other sensors, compress them, and send them to the sink. We therefore consider a WSN deployment environment without obstacles, where all nodes are homogeneous. Following previous studies, the energy consumption for sensor data compression is neglected. When a sensor runs out of energy, operators have to change or recharge its battery. The goal is to maximize the time until the first sensor’s energy is depleted in order to lower the operational costs of the WSN.

**Figure 1:**
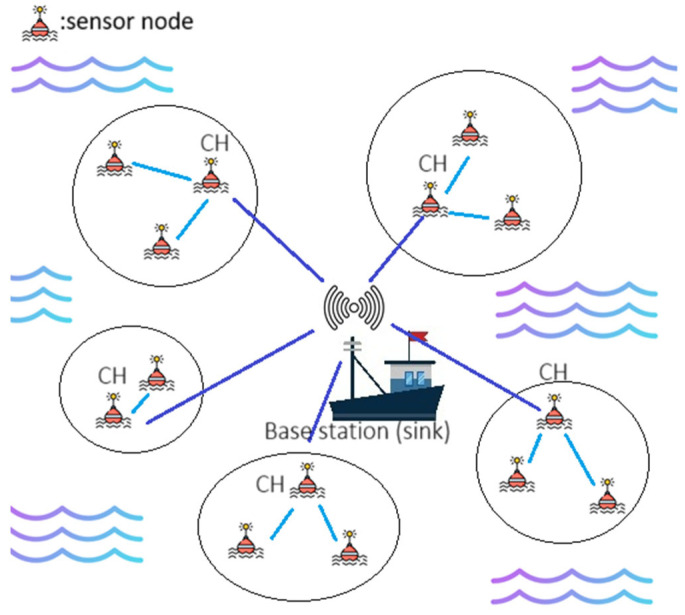
How clustering works in a WSN.

To achieve this goal, we develop a clustering algorithm, which is similar to other popular techniques like LEACH, to reduce sensor energy consumption. We start by selecting CHs at random and then grouping sensors based on how close they are to these CHs. These CHs are responsible for integrating the signals collected by the sensors within their clusters and then transmitting them to the sink. Figure 1 illustrates the operation of the proposed WSN clustering algorithm. Additionally, once a sensor has been selected as a CH, it is temporarily excluded from participating in subsequent CH selections. These processes are necessary to balance energy consumption across the network. The main contribution of this work is the analytical framework, which is based on the model of energy consumption for radio transmission. We present improved formulas for identifying the ideal CH selection zone and the suitable number of clusters within this framework. The following subsections present a detailed explanation of the proposed clustering algorithm.

### 3.1. CH Selection Range

This study aims to prevent the selection of sensors that are too far from the sink as CHs, while also avoiding overuse of nearby sensors as CHs, as discussed in the previous sections. We investigated how much energy the sensor consumed when it transmitted radio signals and when it was operating as a CH to identify the best range for selecting CHs in WSNs. This study uses the energy consumption model for radio transmission by Heinzelman et al. [33], which uses multiple formulas for energy consumption depending on the transmission distance. For convenience, the notation and variables utilized in our calculations are listed in Table 1.

When a radio signal is transmitted over a short distance, the energy required to send the signal is proportional to the square of the distance. However, the energy consumption increases up to the fourth power of the distance when it is longer. The energy consumption of a wireless sensor to receive *l* bits information is

(1) $$E_{r}=lE_{e}+lE_{BF}$$

where $E_{e}$ is the energy consumed by the electronic circuit when transmitting or receiving signals, and $E_{BF}$ is the energy consumed by beam forming. The inclusion of beamforming energy in our model is primarily motivated by the evolving landscape of modern IoT and industrial WSN standards. Contemporary protocols increasingly incorporate support for phased arrays and multi-antenna configurations, such as MIMO systems or compact antenna arrays. Furthermore, the emergence of switched-beam and electronically steerable antennas in high-end sensor nodes has made directional gain a critical factor in energy modeling. By accounting for these advancements, our model provides a more comprehensive and future-proof analysis that reflects the hardware capabilities of next-generation sensor networks.

When the transmission distance *d* exceeds a threshold value, the energy consumption of radio transmission of *l* bits messages is

(2) $$E_{L}(d)=lE_{e}+l\epsilon_{l}d^{4}$$

where $\epsilon_{l}$ is the energy consumed by the amplifier when transmitting messages over a longer distance. If the transmission distance *d* is short, the energy consumption of radio transmission of *l* bit information is

(3) $$E_{S}(d)=lE_{e}+l\epsilon_{s}d^{2}$$

where $\epsilon_{s}$ is the energy consumed by the amplifier when transmitting information over a shorter distance. According to Korteby and Gál [28], the threshold for determining whether to employ the long-distance or short-distance radio transmission model in this study is 87.7 m.

If we evenly distribute *n* sensors and divide them into *k* clusters, each cluster will contain $\frac{n}{k}$ sensors. That is, under the scenario of uniformly distributed sensors, a sensor serving as a CH must bear the additional energy consumption of receiving messages from the other $\frac{n}{k}-1$ sensors. Since the setting of this study is that the selection process of CH will not cause additional energy consumption of the sensor. Therefore, according to Equation (1), when each sensor transmits *l* bits of data to the CH, the extra energy consumption for a sensor acting as a CH is

(4) $$\left(\frac{n}{k}-1\right)E_{r}$$

While preventing sensors farther from the sink from participating in CH selections, we should also avoid excessive energy consumption in other sensors due to too frequently serving as a CH. We will make sure that sensors that are closer to the sink do not use more energy than the energy needed when distant sensors transmit signals to the sink. Otherwise, the proposed strategy could be counterproductive in terms of improving overall energy efficiency.

**Lemma 1.** 
*Suppose the sensors of a WSN are randomly deployed in a square with side length 2X. A circle C is drawn with a radius of r (where r < X) with the center of the square as its center. The probability that a randomly selected sensor falls inside circle C is*$\frac{{\pi r}^{2}}{4X^{2}}$.

**Proof.**  Since the area of the square is $4X^{2}$ and the area of the circle is ${\pi r}^{2}$, the proof of this lemma is trivial. □

**Corollary 1.** 
*If sensors beyond a distance T from the sink are prohibited from participating in CH selections, the expected value of times the remaining eligible sensors actually serve as CHs will become* $\frac{{4X}^{2}}{\pi T^{2}}$*times greater.*

**Proof.**  This corollary can be directly derived from Lemma 1. □

According to Corollary 1 and Equation (4), if sensors that are farther than *T* away from the sink cannot be selected as CHs, the other sensors will have to be CHs more frequently, increasing the energy consumption for receiving information from other sensors by

(5) $$E_{ar}=\frac{{4X}^{2}}{\pi T^{2}}\;\left(\frac{n}{k}-1\right)E_{r}$$

Since Equation (3) applies only to transmission distances shorter than 87.7 m, if the threshold *T* used to exclude sensors from CH election is set greater than 87.7 m, then some CHs whose distance *d* from the sink is greater than *T* will have to use Equation (2) to calculate the energy consumption for transmitting signals to the sink. However, if these sensors are not selected as CHs, their radio transmission energy consumption should be calculated using Equation (3) alone. Hence, when they serve as CHs, they use more energy to transmit radio signals. This extra energy consumption increases with the frequency of their selection as CHs. Each time these sensors serve as CHs, their additional radio transmission energy consumption is denoted as

(6) $${E_{add}=E}_{L}\left(d\right)-E_{S}\left(d_{ic}\right)=l\epsilon_{l}d^{4}-l\epsilon_{s}{\left(d_{ic}\right)}^{2}\;$$

where $d_{ic}\;$ be the average distance between the CH and the sensors in each cluster.

**Lemma 2.** 
Let $\;C_{1}\;$ and $\;C_{2}$ be two concentric circles with radii $r_{1}>r_{2}$. If a point is randomly placed in the area between $C_{1}\;$ and $\;C_{2}$ (the ring-shaped space), then the expected distance of this point from the center is

$$\frac{2\left(r_{1}^{2}+r_{1}r_{2}+r_{2}^{2}\right)}{3\left(r_{1}+r_{2}\right)}.$$

**Proof.**  The radial distance *R* of a point selected at random from the ring satisfies $R\in \left[r_{2},\;r_{1}\right].$ The cumulative distribution function (CDF) for $R\in \left[r_{2},\;r_{1}\right]\;$ is the area of a tiny circle with radius *r*, minus the area of the inner circle $C_{2}$, divided by the total area of the annulus:

$$\frac{\pi r^{2}-\pi r_{2}^{2}}{\pi r_{1}^{2}-\pi r_{2}^{2}}=\frac{r^{2}-r_{2}^{2}}{r_{1}^{2}-r_{2}^{2}}.$$

Taking the derivative of the CDF with respect to *r* gives the probability density function (PDF):

$${\;f}_{R}\left(r\right)=\frac{2r}{r_{1}^{2}-r_{2}^{2}},\;r_{2}<r<r_{1}.$$

Then, the expected value of *R* is:

$$\;\int_{r_{2}}^{r_{1}}rf_{R}\left(r\right)dr=\int_{r_{2}}^{r_{1}}r\frac{2r}{r_{1}^{2}-r_{2}^{2}}dr\;=\frac{2}{r_{1}^{2}-r_{2}^{2}}\int_{r_{2}}^{r_{1}}r^{2}dr\;={\frac{2}{r_{1}^{2}-r_{2}^{2}}\left[\frac{r^{3}}{3}\right]}_{r^{2}}^{r^{1}}\;=\frac{2}{r_{1}^{2}-r_{2}^{2}}\left(\frac{r_{1}^{3}-r_{2}^{3}}{3}\right)\;\;=\frac{2\left(r_{1}-r_{2}\right)\left(r_{1}^{2}+r_{1}r_{2}+r_{2}^{2}\right)}{3\left(r_{1}+r_{2}\right)\left(r_{1}-r_{2}\right)}\;=\frac{2\left(r_{1}^{2}+r_{1}r_{2}+r_{2}^{2}\right)}{3\left(r_{1}+r_{2}\right)}.\;$$

 □

Assuming the sensors are uniformly distributed across the map, consider a threshold *T*. If the sensors are uniformly distributed over the map, among the sensors that are close enough to the sink and can be CHs, a proportion of ${[1-\left(\frac{87.7}{T}\right)}^{2}]$ have a distance exceeding 87.7 m from the sink. According to Lemma 2, the expected distance from these sensors to the sink is

$$d_{r}(T)=\frac{2}{3}\frac{\left({\left(87.7\right)}^{2}+87.7T+T^{2}\right)}{\left(87.7+T\right)},$$

where T>87.7. According Equation (6), for sensors within a distance less than *T* from the sink, the expected additional energy consumption for transmitting signals to the sink when selected as CH is

(7) $$E_{add}=l\epsilon_{l}{d_{r}(T)}^{4}-l\epsilon_{s}{\left(d_{ic}\right)}^{2},where\;T>87.7.$$

**Figure 2:**
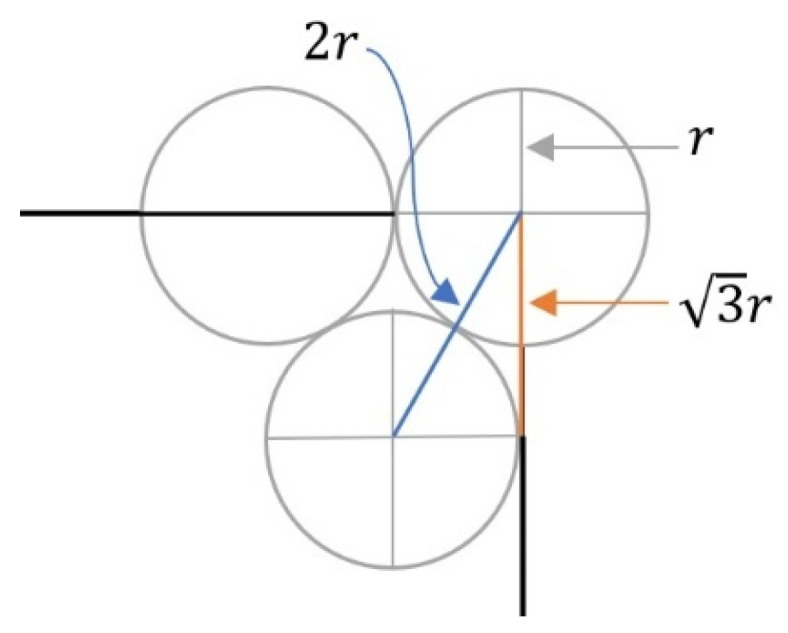
This figure shows how we estimate the radius of *k* circles of the same size that can cover a square area more effectively. When a WSN is divided into *k* clusters, we can use this estimate to determine the average coverage area per cluster. From this, we are able to calculate out how much energy sensors consume to transmit signals to the CH.

To estimate the value of ${\;d}_{ic}$, we consider the problem of determining the smallest radius *d* of *k* circles of the same size that are needed to cover a square with side length *2X*. But there is not a general formula for this problem, so only approximate estimates are available [34]. Since CHs can appear anywhere within the square area, we use the covering method shown in Figure 2 to estimate ${\;d}_{ic}$. In other words,

$$k=\frac{2X}{\frac{\sqrt{3}d_{ic}}{2}}\times \frac{2X}{\frac{\sqrt{3}d_{ic}}{2}},\;\mathrm{and}\;\mathrm{therefore}{\;d}_{ic}=\frac{2X}{\frac{\sqrt{3k}}{2}}=\frac{4X}{\sqrt{3k}}\;.$$

Regarding the potential error introduced by approximating square clusters as disks, we conducted a Monte Carlo simulation to quantify the discrepancy in intra-cluster transmission distances between these two geometries. Our analysis reveals that the average distance error is only 1.7%, which is remarkably small.

Furthermore, the impact of this approximation on our overall energy model is negligible due to the underlying propagation physics of WSNs. In intra-cluster communication (short distances), energy consumption is proportional to the square of the distance; in contrast, long-haul transmissions from CHs to the sink follow a fourth-power path loss. Given that the network’s total energy depletion is dominated by these long-distance transmissions, the minor spatial discrepancy introduced by the disk-based assumption has an insignificant effect on the determination of the optimal number of clusters (k) and the selection threshold (T).

From Equations (5) and (7), it can be inferred that if sensors located farther than *T* from the sink are restricted from participating in CH selection, the expected additional energy consumption incurred by the remaining sensors, due to their increased frequency of serving as CHs, is

$$E_{EVA}=E_{ar}+\left[{1-\left(\frac{87.7}{T}\right)}^{2}\right]E_{add}$$

where T>87.7 and *l* represents the number of bits in the transmitted signal. On the other hand, if T≤87.7, Equation (3) applies to calculating the energy consumption of all sensors selected as CHs when transmitting signals to the sink. Consequently,

(8) $$E_{EVA}=\left\{\begin{array}{c} \;E_{ar}+\left[{1-\left(\frac{87.7}{T}\right)}^{2}\right]E_{add},\;\mathrm{where}\;T>87.7\\ E_{ar},\;\mathrm{where}\;T\leq 87.7 \end{array}\;\right.$$

Next, we estimate the expected energy consumption $E_{EVL}$ for any sensor located at a distance greater than a certain threshold from the sink when transmitting messages to the sink. By setting the distance from the sensor to the sink as a variable and solving the equation $E_{EVL}=E_{EVA}$, an appropriate threshold can be determined to exclude sensors located too far from the sink from participating in CH selection.

We set up the WSN for the experiment on a square map with sides of length 2X, and the sink was in the center of the map. Then, we can derive $E_{EVL}$ by dividing the map into four equally sized square submaps and using only one of the submaps for calculations. Without loss of generality, we only need to consider the submap in the upper-right corner. Let the sink’s coordinates be (0, 0), which corresponds to the lower-left corner of the submap. Sensors are uniformly distributed within the submap. Let the coordinates of an arbitrary sensor be (*x*, *y*), and the distance between the sensor and the sink be denoted as $d=\sqrt{x^{2}+y^{2}}$. For simplicity, we approximate the distance of a sensor within a square to (0,0) by its distance from the center of a disk with radius $r=\sqrt{2}X$ (equal to the diagonal length), in order to calculate the PDF $f_{d}\left(r\right)$ of the distance. To find the expected distance of sensors located more than *T* meters from the receiver, we need to integrate the probability density function of *d* from *T* to the maximum distance $\sqrt{2}X$. The CDF $F_{d}\left(r\right)\;$ gives the probability that the distance *d* is less than or equal to *r*:

$$F_{d}\left(r\right)=P\left(d\leq r\right)=\frac{Area\;of\;the\;region\;where\;\sqrt{x^{2}+y^{2}}\leq r}{Area\;of\;the\;square\;}\;\Rightarrow F_{d}\left(r\right)\approx \frac{\frac{\pi r^{2}}{4}}{x^{2}\;}=\frac{\pi r^{2}}{4x^{2}}$$

The PDF is the derivative of the CDF:

$$f_{d}\left(r\right)=\frac{d}{dr}F_{d}\left(r\right)\;\Rightarrow_{d}\left(r\right)=\frac{d}{dr}\left(\frac{\pi r^{2}}{4x^{2}}\right)=\frac{\pi r}{2x^{2}}$$

Then, the expected value of the distance between the sensors excluded from CH selection and the sink is

$$d_{e}=\frac{\int_{T}^{\sqrt{2}X}r\cdot f_{d}\left(r\right)dr}{\int_{T}^{\sqrt{2}X}f_{d}\left(r\right)dr}=\frac{\int_{T}^{\sqrt{2}X}r\cdot \frac{\pi r}{2x^{2}}dr}{\int_{T}^{\sqrt{2}X}\frac{\pi r}{2x^{2}}dr}=\frac{\int_{T}^{\sqrt{2}X}r^{2}dr}{\int_{T}^{\sqrt{2}X}rdr}=\frac{{\left[\frac{r^{3}}{3}\right]}_{T}^{\sqrt{2}X}}{{\left[\frac{r^{2}}{2}\right]}_{T}^{\sqrt{2}X}}=\frac{\frac{2\sqrt{2}X^{3}-T^{3}}{3}}{\frac{2X-T^{2}}{2}}=\frac{4\sqrt{2}X^{3}-{2T}^{3}}{6X^{2}-3T^{2}}.$$

Therefore, based on Equation (2), the expected value of energy consumption for sensors that are more than *T* distance away from the sink when transmitting *l* bits of signal to the sink can be obtained as $E_{EVL}=E_{L}(d_{e})\;.$

Based on the above discussion, if the value of the threshold *T* leads to $E_{EVA}>E_{EVL}$, it indicates that the sensors participating in CH selection may be forced to serve as CH too many times. We aim to avoid as many long-distance sensors as possible from the participant part in the CH selection. However, the additional energy consumed by the other sensors to become CH must not exceed the energy that the remote sensors would use when transmitting messages directly to the base station. In other words, the maximum value of the threshold *T* should be set such that $E_{EVA}=E_{EVL}$. Equation (8) shows that when *T* > 87.7:

(9) $$E_{ar}+\left[{1-\left(\frac{87.7}{T}\right)}^{2}\right]E_{add}=E_{L}(d_{e})$$

By Equation (9), we can derive a crucial threshold value *T*. This threshold will restrict sensors located beyond a distance of *T* from the sink from participating in CH selections. But Equation (9) does not have an analytical solution for *T*. Hence, we need to substitute parameter values and use numerical methods to estimate *T*. Section 5 will show the approximate values of *T* under different settings and the experimental results.

### 3.2. The Number of CH

To save energy in sensors, the WSN model used in this work does clustering operations at the sink node, which then broadcasts the results to all the sensors. In this architecture, sensors that are selected as CHs consume energy to transmit data to the sink and to receive and integrate data from other sensors in their clusters. In contrast, ordinary sensors only consume energy to transmit data to their allocated CHs.

Assuming that *n* sensors are randomly distributed across the WSN and organized into *k* clusters, let the average energy consumption from all CHs to the sink be $E_{cs}$, based on Equations (1)–(3), the total energy required by the entire WSN to transmit *l* bit of data to the sink can be expressed as

(10) $$kE_{cs}+\left(n-k\right){E_{r}+\left(n-k\right)E}_{S}\left(d_{ic}\right)\;$$

By differentiating Equation (10), the optimal number of cluster heads can be determined.

**Lemma 3.**  *Consider a square region with side length M. A point is uniformly and randomly placed within this square, and the expected Euclidean distance from that point to the center of the square is denoted as *$\frac{M\left(\sqrt{2}+ln\left(1+\sqrt{2}\right)\right)}{6}$.

**Proof.**  Due to the square’s geometric symmetry, the coordinate origin can be shifted to the center of the square without loss of generality. Under this transformation, the center is located at (0, 0), and the square’s domain is defined such that the *x*-coordinate and *y*-coordinate of any point both fall within the range from $\frac{-M}{2}$ to $\frac{M}{2}$. Let $\sqrt{x^{2}+y^{2}}$ represent the Euclidean distance from any arbitrary point to the center, and $\frac{1}{M^{2}}$ represents the probability density of a point uniformly distributed over the square of area $M^{2}$. Since the distance function depends solely on the radial distance from the center and not on the specific quadrant, the integral can be evaluated over the first quadrant and multiplied by four. This leads to the expression for the expected value, denoted as

$$\frac{1}{M^{2}}\int_{\frac{-M}{2}}^{\frac{M}{2}}\int_{\frac{-M}{2}}^{\frac{M}{2}}\sqrt{x^{2}+y^{2}}dxdy\;\;\;\;\;=\frac{4}{M^{2}}\int_{0}^{\frac{M}{2}}\int_{0}^{\frac{M}{2}}\sqrt{x^{2}+y^{2}}dxdy.\;$$

To facilitate integration, a change of variables to polar coordinates is performed in the first quadrant, yielding x=rcosθ, y=rsinθ.  Due to the square-shaped domain, the limits of integration vary with the angle *θ*, and the analysis must consider two cases:

- For $\;\theta \in [0,\;\frac{\pi }{4}]$, the radial limit is determined by the right boundary of the square, yielding $r=\frac{M/2}{\cos\theta }=\frac{M}{2\cos\theta }$.
- For $\;\theta \in [\;\frac{\pi }{4},\;\frac{\pi }{2}]$, the radial limit is determined by the top boundary, yielding $r=\frac{M/2}{\sin\theta }=\frac{M}{2\sin\theta }$.

Next, by applying the Jacobian matrix [35]

$$J=\left[\begin{array}{c} \frac{\partial x}{\partial r}\;\frac{\partial x}{\partial \theta }\\ \frac{\partial y}{\partial r}\;\frac{\partial y}{\partial \theta } \end{array}\right]=\left[\begin{array}{c} cos\theta -rsin\theta \\ sin\theta \;rcos\;\theta \end{array}\right]\;\Rightarrow \det J=r\cos^{2}\theta +r\sin^{2}\theta =r,$$

we obtain dxdy=rdrdθ. Consequently, the integral over the first quadrant is expressed as

$$\frac{4}{M^{2}}\left(\int_{0}^{\frac{\pi }{4}}\int_{0}^{\frac{M}{2\cos\theta }}r^{2}drd\theta +\int_{\frac{\pi }{4}}^{\frac{\pi }{2}}\int_{0}^{\frac{M}{2\cos\theta }}r^{2}drd\theta \;\right)\;=\frac{4}{M^{2}}\left(\int_{0}^{\frac{\pi }{4}}\frac{1}{3}{\left(\frac{M}{2\cos\theta }\right)}^{3}d\theta +\int_{\frac{\pi }{4}}^{\frac{\pi }{2}}\frac{1}{3}{\left(\frac{M}{2\sin\theta }\right)}^{3}d\theta \right)\;=\frac{M}{6}\int_{0}^{\frac{\pi }{4}}\sec^{3}\theta d\theta +\int_{\frac{\pi }{4}}^{\frac{\pi }{2}}\csc^{3}\theta d\theta \;=\frac{M}{6}\int_{0}^{\frac{\pi }{4}}\sec^{3}\theta d\theta +\int_{\frac{\pi }{4}}^{\frac{\pi }{2}}\csc^{3}\theta d\theta \;={\frac{M}{6}\left[\frac{\sec\theta \tan\theta +\ln\left|\sec\theta +\tan\theta \right|}{2}\right]}_{0}^{\frac{\pi }{4}}+\;\frac{M}{6}\;{\left[\frac{-\csc\theta \cot\theta +\ln\left|\csc\theta -\cot\theta \right|}{2}\right]}_{\frac{\pi }{4}}^{\frac{\pi }{2}}\;=\frac{M}{6}\left(\frac{\left(\sqrt{2}+ln\left(1+\sqrt{2}\right)\right)}{2}+\frac{\left(\sqrt{2}-ln\left(\sqrt{2}-1\right)\right)}{2}\right)\;=\frac{M}{6}\left(\frac{\left(\sqrt{2}+ln\left(1+\sqrt{2}\right)\right)}{2}+\frac{\left(\sqrt{2}+ln\left(\sqrt{2}+1\right)\right)}{2}\right)\;=\frac{M\left(\sqrt{2}+ln\left(1+\sqrt{2}\right)\right)}{6}$$

 □

**Lemma 4.**  *Draw a circle with radius R and randomly place a point inside it. The expected distance from this point to the center of the circle is*$\frac{2R}{3}$.

**Proof.**  Assume a circular region centered at the origin (0, 0) with radius *R*. The location of a randomly selected point within this circle can be described using polar coordinates (*r*, *θ*), where *r* denotes the radial distance from the origin, and *θ* is the angle with respect to the horizontal axis. Given that the points are uniformly distributed over the area of the circle, the probability of a point falling within any region is directly proportional to the area of that region. The total area of the circle is $A=\pi R^{2}$.

To determine the probability distribution of the radial distance *r*, consider a thin annular ring centered at the origin with inner radius *r* and outer radius *r* + *dr*. The CDF of *r* is given by

$$\frac{\pi r^{2}}{\pi R^{2}}=\frac{r^{2}}{R^{2}}.$$

Hence, differentiating the CDF yields the PDF *f*(*r*):

$$f\left(r\right)=\frac{d}{dr}\left(\frac{r^{2}}{R^{2}}\right)=\frac{2r}{R^{2}},\;0\leq r\leq R.$$

The expected value of the distance from a randomly chosen point to the center, *E*[*r*], can thus be computed as:

$$E\left[r\right]=\int_{0}^{R}rf\left(r\right)dr=\int_{0}^{R}r\frac{2r}{R^{2}}dr=\frac{2}{R^{2}}\int_{0}^{R}r^{2}dr=\frac{2R}{3}.\;$$

 □

To estimate the value of $E_{cs}$ within a square region, it is necessary to consider two distinct cases: when the CH’s distance to the sink is less than 87.7 m, and when it is greater than or equal to 87.7 m. This difference comes from the fact that each situation has a different model for how much energy it consumes. To derive an approximate expression for the CH’s energy consumption, we must first estimate the expected distance from a CH to the sink under both conditions.

According to Lemma 4, when several sensors are uniformly distributed within a circular region of radius 87.7 m, the expected distance from a sensor to the center of the circle is denoted as

$$d_{i}=\frac{2\times 87.7}{3}=\frac{175.4}{3}.$$

In contrast, consider a square region with side length *M* greater than 87.7×2=175.4 m. If sensors are randomly deployed within this square, and a circle *C* of radius 87.7 m is drawn with the square’s center as its center, then evaluating the expected distance from sensors located outside circle *C* (but still within the square) to the center becomes analytically intractable. To address this challenge, we approximate the region outside circle *C* by substituting the square with a circular region, thereby simplifying the analysis. Leveraging Lemma 2, we approximate the expected distance in this case as

$$d_{r}\left(\frac{\sqrt{2}m}{2}\right)=\frac{2\left(87.7^{2}+87.7\cdot \frac{\sqrt{2}m}{2}+\frac{m^{2}}{2}\right)}{3\left(87.7+\frac{\sqrt{2}m}{2}\right)}.$$

Consider a square region with side length *M*, which is uniformly partitioned into *k* smaller, equally sized squares. Let $S_{1},S_{2},\ldots ,S_{k}$ denote one such smaller square, with each having a side length of $\frac{M}{\sqrt{k}}$. Based on the result established in Lemma 3, the expected distance between two points that are independently and uniformly distributed within square $S_{1},S_{2},\ldots ,S_{k}$ is given by $d_{ic}=\frac{M\left(\sqrt{2}+ln\left(1+\sqrt{2}\right)\right)}{6\sqrt{k}}$.

**Corollary 2.** 
*Consider a WSN deployed over a square region with side length M, which is evenly divided into k smaller squares, and the sink node is positioned at the center of the square. When sensors are randomly distributed throughout this area, the expression in Equation (10) can be approximated by*

(11) $$k\left(E_{cs}\right)+\left(n-k\right){(E}_{r}+E_{S}\left(d_{ic}\right)),$$

*where* $E_{cs}=\frac{{{\pi 87.7}^{2}\epsilon }_{s}}{M^{2}}E_{S}\left(d_{i}\right)+\frac{{M^{2}-\pi 87.7}^{2}}{M^{2}}E_{L}\left(d_{r}\left(\frac{\sqrt{2}M}{2}\right)\right)$.

**Proof.**  According to the energy consumption model, sensors situated within 87.7 m of the sink follow the expression in Equation (3), while those located at distances equal to or greater than 87.7 m follow Equation (2). The percentage of sensors in the WSN with a distance to the sink less than T is $\;\frac{87.7^{2}}{M^{2}}$; therefore, the percentage of sensors with a distance to the sink greater than or equal to *T* is $\frac{{L^{2}-87.7}^{2}}{M^{2}}$. Substituting this result into Equation (10) completes the proof. □

Equation (11) shows the approximate total energy needed for the whole WSN to transmit an *l*-bit signal when the sensors in a WSN are split into an average of *k* clusters. We may discover the condition that consumes the least energy by dividing this equation by *l*, taking the derivative with respect to *k*, and setting the derivative to zero. This approach lets us figure out the optimal number of clusters in theory, assuming the sensors are uniformly and randomly distributed. We can find Equation (12) by dividing Equation (11) by *l*, taking the derivative with respect to *k*, and setting the derivative to zero:

(12) $$\frac{E_{cs}}{l}-2E_{e}-E_{BF}-\frac{\epsilon_{s}{nd_{ic}}^{2}}{k}=0$$

By substituting $d_{ic}=\frac{M\left(\sqrt{2}+ln\left(1+\sqrt{2}\right)\right)}{6\sqrt{k}}$ into Equation (12) and differentiating with respect to *k*, we obtain

(13) $$k=\sqrt{\frac{\epsilon_{s}{n\left(M\left(\sqrt{2}+ln\left(1+\sqrt{2}\right)\right)\right)}^{2}}{36\left[E_{cs}-{2E}_{e}-E_{BF}\right]}.}\;$$

Accordingly, the clustering algorithm proposed in this work adopts Equation (13) to determine the number of clusters used to partition the WSN

## 4. Proposed Clustering Algorithm and Hybrid Model

We introduce a clustering strategy that restricts sensors in specific areas from participating in the CH election process. Using the energy consumption model, we found a threshold distance, which is the solution to Equation (9), to figure out the optimal radius for the CH selection zone. Sensors located beyond this threshold are excluded from the CH selection process. Furthermore, Equation (13) was used to determine the number of clusters. The suggested HEOCP combines this clustering strategy with a ResNet-50 deep learning model that employs a GA. This allows ResNet-50 to average out the GA’s noisy predictions and provide an effective, intelligent approach for CH selection.

Furthermore, as the calculation of Equation (13) explicitly incorporates both the side length of the square deployment area and the total sensor count, our framework is inherently capable of adapting to diverse sensor densities. This design ensures that HEOCP can accommodate a wide range of application scenarios with varying node distributions without requiring manual recalibration.

Whereas traditional approaches rely on heuristic algorithms to tune parameters for deep learning models, we propose a novel teacher-student surrogate framework in which deep learning models learn directly from the outputs of heuristic algorithms. To the best of our knowledge, this approach represents a novel contribution to the wireless sensor network domain.

**Figure 3:**
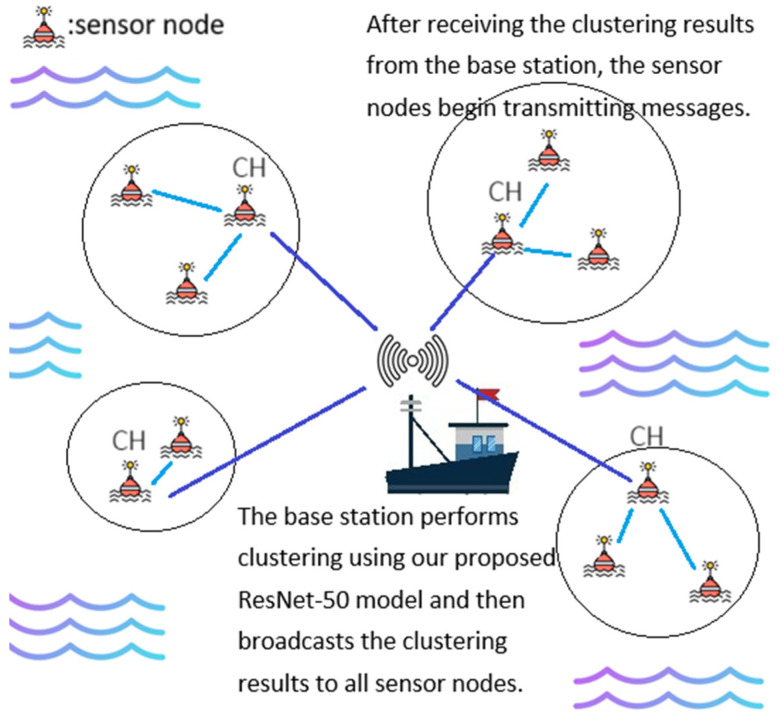
The operational mode of the HEOCP.

As previously noted, our proposed HEOCP clustering protocol is executed exclusively at the sink node, which is equipped with high-performance computing capabilities. Once the clustering decisions are finalized, the sink simply broadcasts the resulting configuration back to the individual sensors in the WSN. Consequently, the HEOCP inference process does not deplete the limited energy reserves of the sensor nodes. Furthermore, since the image encoding and processing occur locally within the sink, the generation of these images incurs no wireless communication overhead across the network. The operational workflow of HEOCP is illustrated in Figure 3.

We convert the deployment of the WSN into an image and use a deep learning model to learn the prediction results of GA. The goal is to enable the deep learning model to simultaneously capture the individual status of each sensor, the local topology of each sensor (neighbor node distribution), and the global structure of the WSN (sink location and boundary regions). It is challenging for basic convolutional neural networks (CNNs) to capture both the local topology and the global WSN structure at the same time since they have small receptive fields and a limited number of nonlinear layers.

In our study, ResNet-50 is employed to learn the CH selection behavior of the GA within dynamic and complex WSN environments. The GA evaluates a variety of parameters, such as node locations and residual energy levels, to determine optimal CH sets. These selected CH combinations serve as labeled data for training ResNet-50. ResNet-50 has sufficient depth and an appropriate residual structure to provide both a large receptive field and strong nonlinearity. This enables the model to learn optimal paths from nodes to the sink, identify energy distribution patterns across the network, and determine which nodes to avoid in the current round, all while avoiding excessive exploration that could capture a bunch of complex patterns unrelated to selecting CH. Owing to its depth, ResNet-50 can capture intricate patterns, enabling it to approximate the GA’s selection behavior with high accuracy.

This integration allows the network to perform CH selection in real-time without the need for re-running the computationally expensive GA. Consequently, ResNet-50 facilitates faster clustering while preserving the quality of GA-generated configurations, thereby enhancing the overall efficiency and energy management of the WSN.

We do not choose deeper ResNet models such as ResNet-101 because the output of GA is only an approximate optimal and heuristic solution. Since GA itself introduces randomness and heuristic bias, the labels derived from its output are inherently noisy. Using ResNet-101, the model might learn too many patterns that are not useful for CH selection and become too sensitive to noise. Moreover, the dataset is still relatively small for a model with the number of parameters in ResNet-101, no matter how many WSN scenarios and rounds there are. Therefore, eventually, we decide to employ ResNet-50 to learn from GA’s output.

The experimental settings are explained in the rest of this subsection and in Section 5. The experimental findings show that ResNet-50 performs better than the alternatives considered.

**Figure 4:**
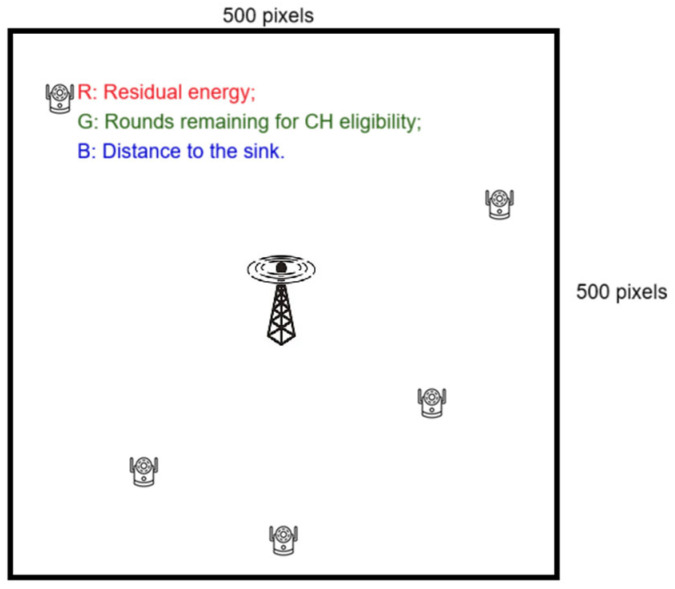
Convert the WSN deployment environment into an image with sensors represented as pixels and their data recorded in RGB channels.

To train with ResNet-50, we deployed the WSN on a two-dimensional plane with both x- and y-coordinates ranging from 0 to 499, and the sink positioned at the center of this area. In each of the ten simulation scenarios, 100 sensors are randomly distributed, with integer-valued coordinates. The clustering algorithm utilized by our proposed HEOCP is simulated, and a GA is used to determine the optimal CH set. The GA’s output is then used to generate the training dataset. This setup allows the WSN environment to be represented as a 500 × 500-pixel image, where each sensor occupies a single pixel. For each pixel corresponding to a sensor, three features are encoded into the red, green, and blue (RGB) channels: the sensor’s residual energy, the number of remaining rounds before it becomes eligible for CH selection, and its Euclidean distance from the sink. Pixels not associated with sensors are assigned RGB values of zero, resulting in a complete 500 × 500 image representation of the WSN environment, as illustrated in Figure 4.

We generated training images for two sensor types: “suitable to be CH” and “unsuitable to be CH.” These labeled images are then used to train a ResNet-50 model, enabling it to learn the decision-making patterns of the GA. While mapping the WSN to a bitmap representation inherently introduces sparsity (where most pixels remain unoccupied), this design choice is intentional. Our primary motivation is to provide a comprehensive global scene view, which allows CNN-based architectures to leverage spatial inductive biases to capture both local neighborhood patterns and global structural features, such as boundary effects and sink node positioning.

By adopting a fixed-resolution ‘scene view,’ we maintain a constant input dimension that bypasses the challenges of variable-length data, allowing us to leverage highly optimized, off-the-shelf CNN implementations. A key advantage of this representation is that it eliminates the need for architectural redesigns as node density increases. Whether the deployment consists of 100 or 1000 nodes, the input remains a consistent grid; the user simply updates the pixel-wise occupancy or intensity values. This ensures a uniform model architecture across various deployment scales, making our approach particularly robust for high-density IoT applications, such as those found in complex urban environments. To the best of our knowledge, prior research has not translated WSN geometry into fixed-resolution images to maintain spatial relationships as we have done.

Furthermore, the core objective of our approach is to replace the iterative, computationally expensive GA optimization with a single forward inference pass during runtime. This significantly reduces online computational overhead. It is also important to note that the ResNet-based inference is executed at the sink node, which is equipped with high-performance computing resources; therefore, this process does not deplete the limited energy reserves of the individual sensor nodes. To further evaluate the trade-offs between computational cost and performance, we have detailed the corresponding inference latency and network lifetime metrics in Section 5.

**Figure 5:**
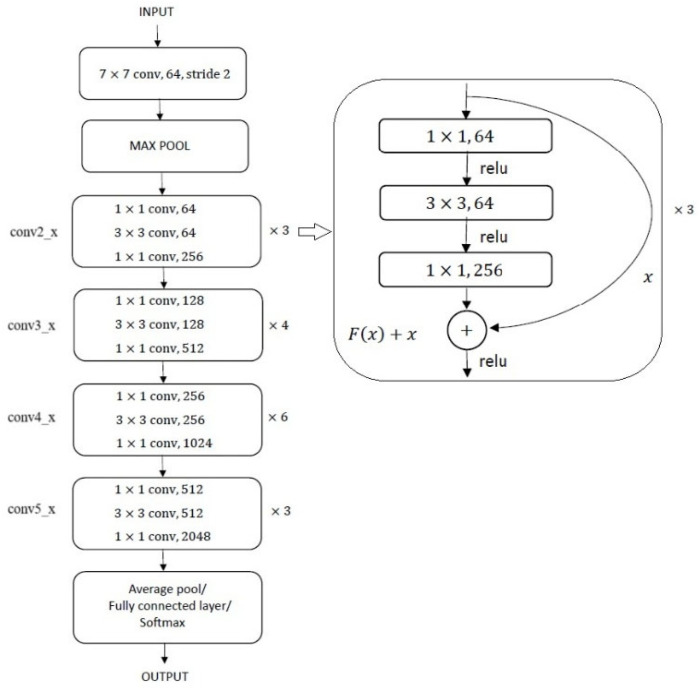
The figure illustrates the structure of ResNet-50. The construction of a single residual block in the first residual stage is shown to the right of the figure. Other residual blocks are similar, but their convolutional layers are designed uniquely at different stages.

ResNet-50 is a deep convolutional neural network comprising 50 layers, organized into an initial convolution and max-pooling stage followed by four major residual block stages: conv2_*x* through conv5_*x*. Each block consists of multiple residual units that enable efficient gradient flow during training. We present the structure of ResNet-50 in Figure 5.

We trained the deep learning model with 10,288 images created using a genetic algorithm. 5144 of these images showed sensors that were suitable to be CH, and the other 5144 showed sensors that were unsuitable to be CH. We ran simulations on 10 different WSN topologies until the first sensor ran out of energy to acquire these images. To assess the soundness and generalizability of our choice to use the ResNet 50 model for learning the GA’s output, we randomly placed 100 sensors within a square area, each side measuring 300 m. Afterward, we implemented the CNN, ResNet 50, and ResNet 101 models to learn the output of the GA-selected CHs. We then applied these learned models to the LEACH protocol. The results, presented in Table 2, imply that ResNet 50 performed better.

While the basic CNN baseline (325.1 rounds) shows competitive performance, it exhibits the following limitations:

1. Limited Receptive Field: Basic CNNs with 3–5 layers struggle to capture global WSN topology, especially in large-scale deployments (500 × 500 m).
2. Gradient Flow Issues: Without residual connections, deeper CNN variants suffer from vanishing gradients, preventing effective training on our 10,288-image dataset.

The 4.1% improvement of ResNet-50 over CNN, though modest, represents approximately 13 additional operational rounds—a significant extension considering WSN energy constraints.

In the next section, to verify the effectiveness of our proposed HEOCP, we also generated 10,288 images as a training set, as described above. In the experiment in Section 5, the largest WSN environment was a square space with 500-meter sides. Hence, the model’s input image size was set at 500×500 pixels. The maximum number of training epochs was set to 30.

The computer that was used for training had a 2.4 GHz Intel Xeon Gold 6240R processor, an NVIDIA GeForce RTX 4090 graphics card, and 512 GB of RAM. Ubuntu 22.04.4 LTS was the operating system. The dataset was divided into two parts: training and validation, with 80% going to training and 20% going to validation.

**Figure 6:**
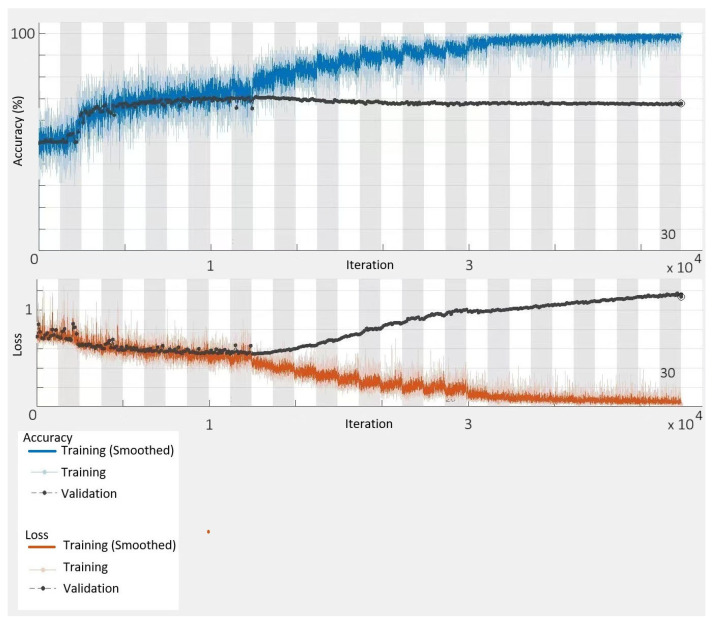
The training performance of ResNet-50.

The training was performed on the GPU with a mini-batch size of 32 and a starting learning rate of 0.001. Every 100 iterations, the model’s performance was checked on the validation set. The data was randomly shuffled at the start of each epoch to make training more stable. A piecewise learning rate decay strategy was also used, and the training process could be seen in real time. Figure 6 shows how the model’s accuracy and loss changed over time throughout training. 

**Figure 7:**
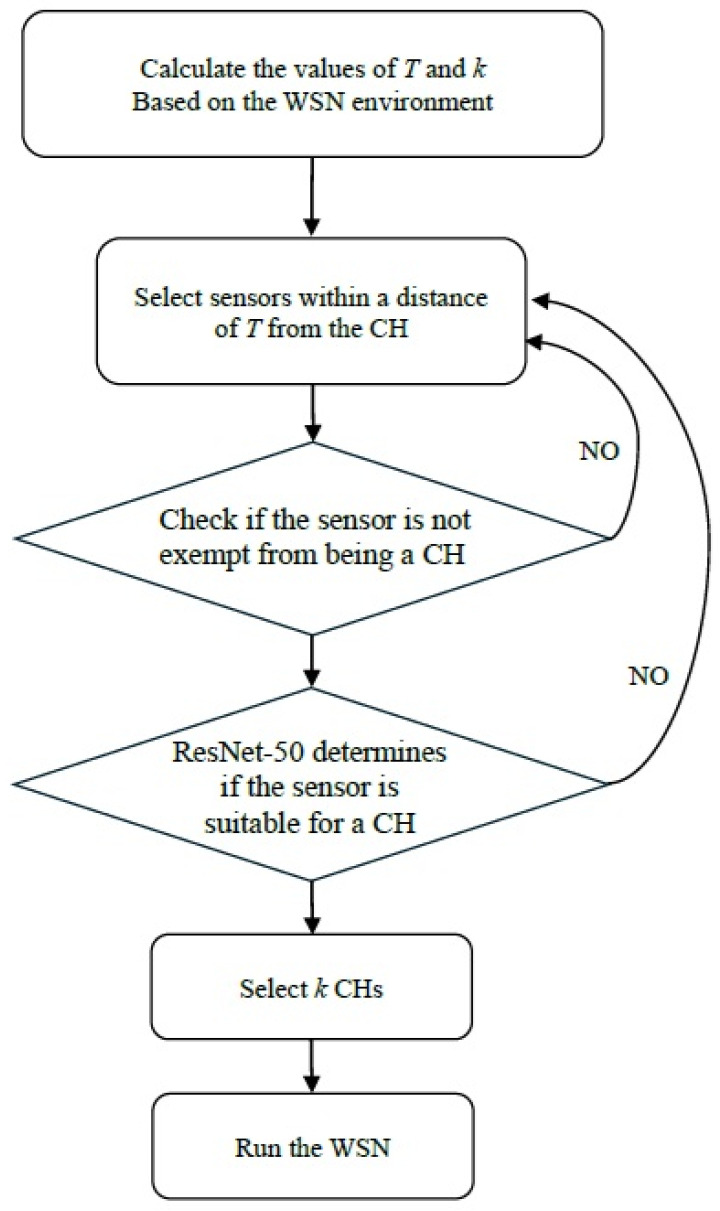
The flowchart of the HEOCP.

The flowchart for HEOCP is illustrated in Figure 7. To evaluate the temporal efficiency of the proposed HEOCP framework, we conducted a comparative analysis between the GA and our trained ResNet-50 model across ten distinct map topologies with side lengths of 300 m, 400 m, and 500 m. In each scenario, both approaches were utilized to perform CH selection for the WSN. The average execution times are recorded in Table 3. The experimental results demonstrate that HEOCP significantly reduces computational overhead, underscoring its suitability for practical deployment in resource-constrained WSN environments.

As shown in Table 3, replacing iterative GA optimization with a single forward pass reduces the per-round CH-selection latency from tens to hundreds of seconds (GA) to around 2 seconds (HEOCP), supporting real-time operation at the sink side. If further reduction in latency or memory footprint is required for edge-only deployments, standard model compression techniques—including knowledge distillation (e.g., ResNet-50 → a lightweight student), post-training INT8 quantization, and structured channel pruning—can be applied. In addition, because the WSN-to-image representation is sparse, practical acceleration can also be achieved by reducing input resolution or employing region-focused inference, which directly reduces the computational cost.

In summary, the HEOCP leverages the strengths of both genetic algorithms and deep convolutional neural networks. By constraining CH selection to energy-efficient regions and optimizing the number of clusters, the HEOCP enhances network longevity. The GA searches for high-quality CH configurations through evolutionary optimization. Meanwhile, ResNet-50 learns and stably reproduces these strategies by understanding the entire WSN deployment environment. This process mitigates the noise introduced by the GA’s stochastic nature, ultimately enabling efficient real-time deployment.

## 5. Experimental Results

In this section, we provide the experimental findings of our work. The experiment was carried out by simulating a WSN with MATLAB R2023a (MathWorks, Natick, MA, USA). As we mentioned in Section 4, our research objective is to delay the appearance of sensors that are insufficiently powered in WSN. More specifically, a protocol is better if it achieves more execution rounds in the WSN before the first sensor runs out of energy (First Node Dead, FND). We will compare our proposed HEOCP to current popular random clustering algorithms. We also compare HEOCP to the Grey Wolf Optimization (GWO) and Particle Swarm Optimization (PSO) algorithms, as well as some new genetic algorithm-based clustering methods, to show the superior performance of our trained ResNet-50 in extending the lifetime of the WSNs.

To ensure the robustness of our findings, we established a controlled experimental setup. The simulation parameters are as follows:

In Table 4, the amounts for radio transmission consumption and communication parameters (such as path-loss exponent, circuit consumption of energy, and power-amplification coefficient) are all based on [28]. The coordinates of each sensor are randomly generated pairs of integers, and the framework automatically calculates the actual distance between any two sensors based on their relative coordinates.

We plug the values from Table 4 into Equations (9) and (13) to determine the threshold value *T* and the number of clusters *k* for different environment sizes. A sensor cannot be selected as a CH if its distance from the sink is greater than *T*. We conducted experiments on square environment maps with side lengths of 300 m, 400 m, and 500 m, respectively. We found the values of *T* and *k* using Equations (9) and (13). Next, we will employ the values from Table 4 for clustering in the WSN.

Table 5 indicates that the number of clusters determined using the energy consumption calculation decreases when the WSN’s deployment range increases. This phenomenon is because the amount of energy used for transmitting radio signals is proportional to the distance they travel by a quadratic or quartic polynomial. Subsequently, when the map size gets larger, the amount of energy a CH requires to transmit signals to the sink increases quickly, and the gap between this value and the amount of energy needed to receive signals rapidly widens. As a result, the formula’s outcomes tend to prefer fewer CHs. The WSN model we selected has a connection to this issue. If we adopted a model where it took a lot of energy for a CH to collect and compress data, the trend might be different. Furthermore, we must also consider that as the map size increases, if the number of clusters is too small as the map size grows, it could cause sensors within a cluster to have to transmit signals over too long of a distance, which would not be compatible with the settings for intra-cluster transmission energy use in the derivation of Equation (13). If we were to cover the WSN deployment region with *k* circles of the same size, we can identify the lower bound of the radius, $r_{l}$, of each circle for different values of *k*. Unfortunately, there is no general formula that can determine $r_{l}$ directly by the value of *k*. If the determined $r_{l}$ exceeds 87.7 m, it does not match the conditions of the derived Equation (13).

**Figure 8:**
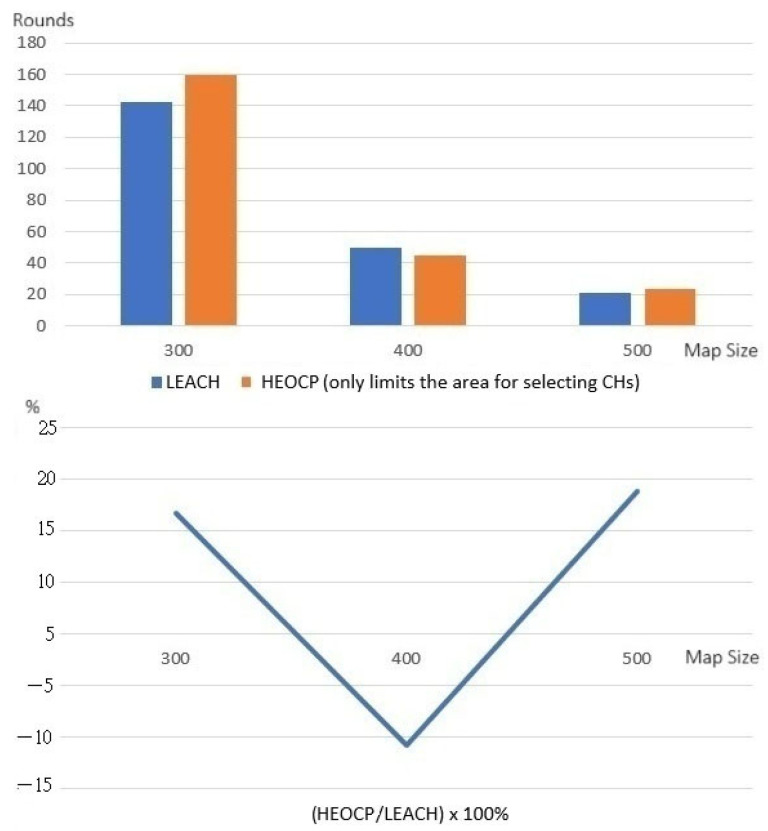
Deploying 100 sensors in all maps to test if narrowing the CH selection area improves clustering.

We tested our idea for reducing the CH selection area on the popular clustering algorithm LEACH to see if it worked. Figure 8 displays the results. The data corresponding to HEOCP represents the outcome of running LEACH with the rule that only sensors within a distance T of the sink could be selected as CHs.

**Figure 9:**
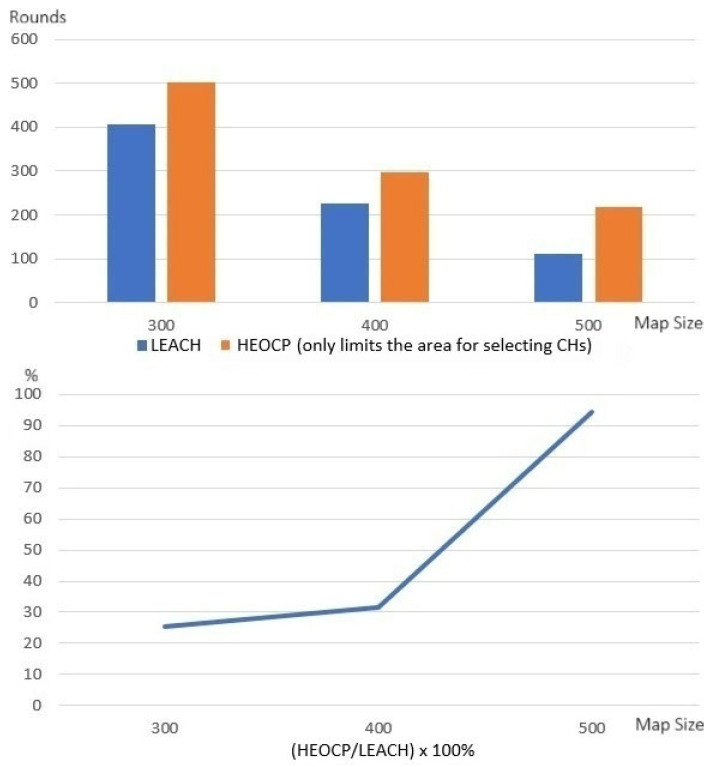
Increasing the number of sensors in the map to verify whether narrowing the area for CH selection can improve clustering.

The results from the experiment show that our strategy for limiting the CH selection area significantly improves LEACH’s performance, except for maps with a side length of 400 m. In the 400 m map experiment, our method’s performance was slightly lower than LEACH’s, which could be because there were not enough sensors. If the sensor density in the map is too low, the probability of locating severely uneven sensor distributions increases. Hence, we did further tests with more sensors. We placed 450, 800, and 1250 sensors in maps with side lengths of 300, 400, and 500 m and ran the experiments again. Figure 9 shows the results. These results show that our approach always works better than LEACH, regardless of the map’s side length. Therefore, we are confident that our idea of limiting the area where the cluster head can be selected will enable the clustering mechanism to work better as long as the sensor density on the map is not too low and the distribution is not too uneven.

**Figure 10:**
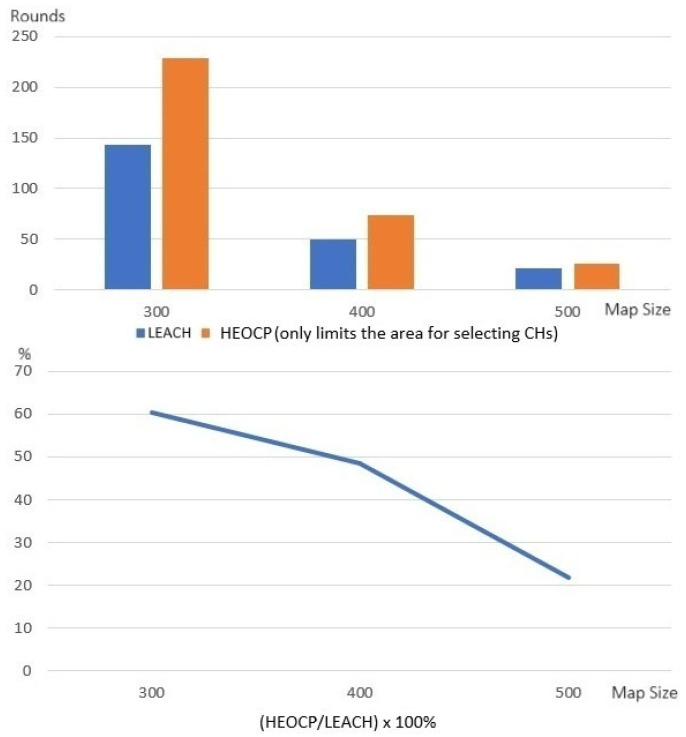
The performance of extending the lifetime of WSN is evaluated according to the different cluster number limitations of HEOCP and LEACH.

Next, we conducted experiments to ensure that the proposed HEOCP’s cluster count limitation also benefits the lifetime of the WSNs. Similarly, we run the LEACH protocol but determine the number of clusters using Equation (13) and compare it to the standard LEACH. Figure 10 shows that the limit on the number of clusters set by HEOCP does actually greatly lengthen the lifetime of the WSN. We believe that our proposed clustering algorithm, which uses the number of sensors and the size of the WSN deployment environment to determine how many clusters there should be, is more effective. The experiments mentioned above examined the performance of HEOCP’s two major mechanisms: limiting the range of CH selection and dynamically determining how many CHs there should be. Next, we evaluate the whole HEOCP clustering algorithm, which limits the CH selection range and dynamically sets the number of CHs, to verify its effectiveness in extending WSN lifetime.

**Figure 11:**
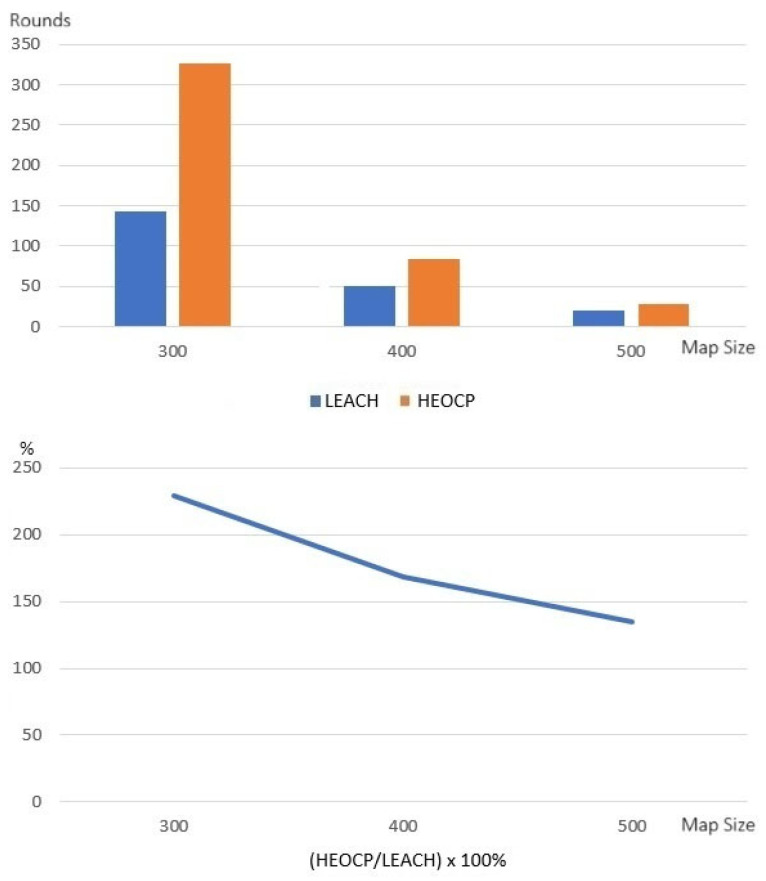
The results of the performance comparison between HEOCP and LEACH.

Figure 11 displays the efficacy of the HEOCP clustering algorithm across ten randomly generated WSN environments. It can greatly increase the lifetime of WSNs compared to the currently used LEACH, especially in smaller WSN deployment environments. This verifies that our strategy of using the radio transmission energy consumption formula to develop a model and then clustering it is beneficial and can help guide future study in this area.

**Figure 12:**
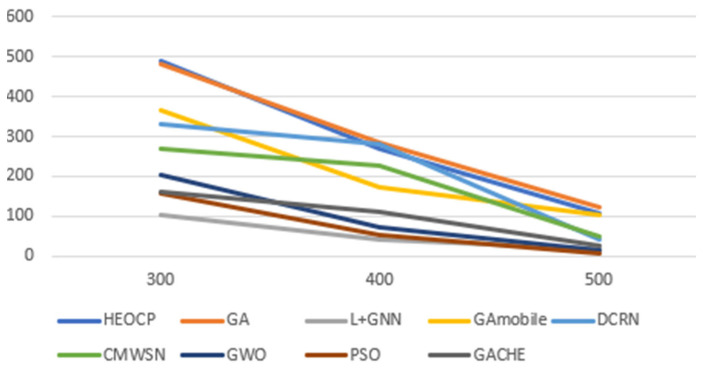
Comparative Evaluation of HEOCP and Baseline Methods.

In the end, we compared the proposed HEOCP framework to GWO, PSO, the GA we employed for training, and three other GA-based clustering methods from relevant research (DCRN [19], GACHE [20], CMWSN [36]). In addition to the aforementioned models, we extended our comparative analysis by incorporating MobileNetV3 [37] as an alternative backbone for learning GA-generated labels. Furthermore, we implemented a novel architecture that integrates Graph Neural Networks (GNN) with the LEACH protocol [38] to serve as a contemporary baseline. The performance metrics for both models have been integrated into our experimental results to provide a comprehensive evaluation against the proposed HEOCP framework. We did this comparison by randomly placing 100 sensors in square deployment scenarios with side lengths of 300 m, 400 m, and 500 m, respectively. Figure 12 presents the averaged results from three independent simulation trials. The empirical evidence demonstrates that our proposed HEOCP framework is highly effective in extending the operational longevity of WSNs. Notably, while its performance closely approximates that of the GA it was trained to emulate, HEOCP achieves this with significantly greater computational efficiency. This drastic reduction in execution time renders HEOCP particularly well-suited for time-sensitive WSN deployments, where real-time responsiveness and near-optimal resource management are paramount.

To evaluate the generalizability of HEOCP, we conducted experiments in a non-uniform deployment scenario. Specifically, we modified the 300 × 300 m, 400 × 400 m, and 500 × 500 m square maps by removing a 30 × 30 m square from each of the four corners, creating a cross-shaped topology. Furthermore, the sink node was relocated from the center of the map to the left boundary. This setup allowed us to test the framework under conditions characterized by uneven node distribution and an off-center sink placement.

**Figure 13:**
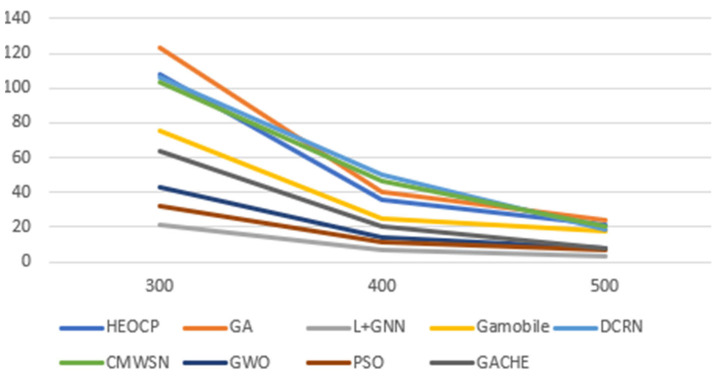
Performance evaluation across cross-shaped network topologies.

The experimental results are illustrated in Figure 13. As observed, shifting the sink to the left boundary of the map significantly increased the communication distance for sensors located in the right hemisphere, resulting in a marked reduction in overall WSN longevity. Furthermore, because these experimental conditions deviate from the theoretical assumptions used in our initial formula derivation, the GA model learned by HEOCP did not yield the absolute optimal performance in every scenario. Specifically, alternative GA variants exhibited slightly superior results in the *m* map configuration.

**Figure 14:**
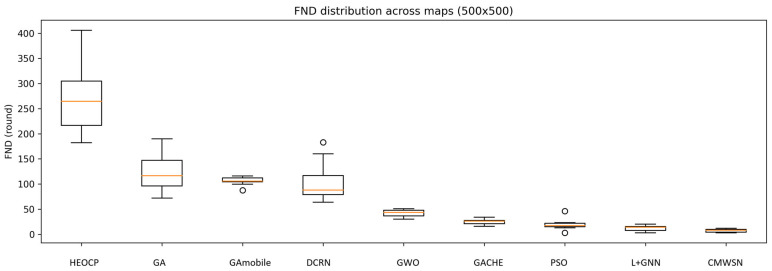
Box plot analysis of the 300 m scale experiments.

**Figure 15:**
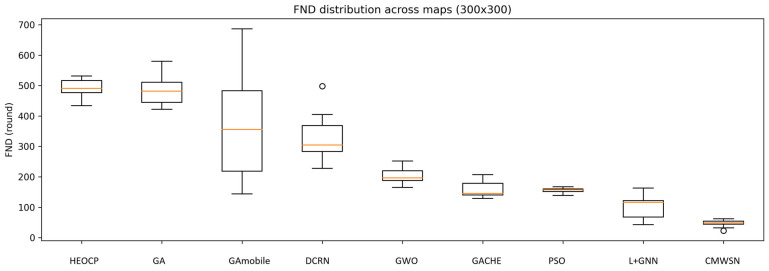
Box plot analysis of the 400 m scale experiments.

**Figure 16:**
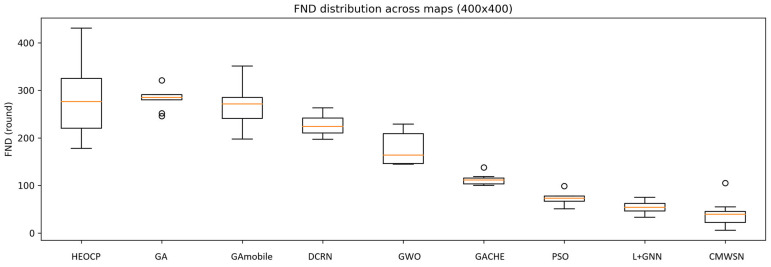
Box plot analysis of the 500 m scale experiments.

To comprehensively evaluate the performance and stability of HEOCP relative to existing methods, we conducted three independent experimental trials across three distinct map scales. The results are summarized in Table 6, Table 7 and Table 8 and Figure 14, Figure 15 and Figure 16. To rigorously assess robustness, we employed the Coefficient of Variation (CV); our results indicate that HEOCP’s fluctuations remain well-controlled relative to its performance magnitude across all map sizes.

The Interquartile Range (IQR) provides an additional perspective on stability by focusing on the central 50% of the distribution, effectively mitigating the influence of outliers. HEOCP maintains competitive IQR values in all scenarios—most notably on the 500 m edge-length maps, where its IQR is significantly narrower than those of most baseline methods. This demonstrates that HEOCP’s typical execution results are tightly clustered around the median. Furthermore, the narrow confidence intervals indicate a high degree of precision in our estimation of HEOCP’s expected performance. The 10th percentile (P10) serves as a conservative performance lower bound; HEOCP’s P10 consistently remains favorable compared to the overall distributions of competing methods, suggesting that sub-optimal outcomes are rare outliers rather than common occurrences.

These characteristics underscore HEOCP’s suitability for real-world deployment scenarios that demand both high expected performance and predictable behavior. While no algorithm can simultaneously maximize performance and minimize all variability metrics, HEOCP occupies an ideal position within this trade-off space—delivering superior average performance while maintaining stability metrics that are comparable to, or better than, methods operating at lower performance tiers.

Overall, the experimental results demonstrate that HEOCP offers greater optimization efficacy compared to alternative methods. Across diverse map environments, it consistently achieves either the longest or near-optimal average network lifetime while maintaining high computational efficiency. These findings suggest that HEOCP is a highly effective approach for prolonging the operational longevity of WSNs, offering a compelling balance between performance and real-time feasibility.

Next, we investigate the sensitivity of the HEOCP framework to label noise. In our proposed architecture, supervisory labels are generated via a GA. Due to the inherent stochasticity of the GA, we define “label noise” as the inconsistency between labels produced by executing the GA multiple times on the same state with different random seeds.

To quantify this, we sampled 50 state snapshots from the training topologies (5 snapshots per topology) and executed the GA five times for each snapshot using distinct seeds. The degree of overlap among the resulting CH sets was measured using pairwise Jaccard similarity, the results of which are summarized in Table 9.

Our experimental results indicate non-trivial variability across multiple GA runs. To evaluate the framework’s sensitivity to this pseudo-label noise, we trained two learning models with identical architectures:

1. A baseline model, trained using labels from a single GA execution.
2. An aggregated-label model, trained using labels determined by a majority vote across five GA runs.

**Figure 17:**
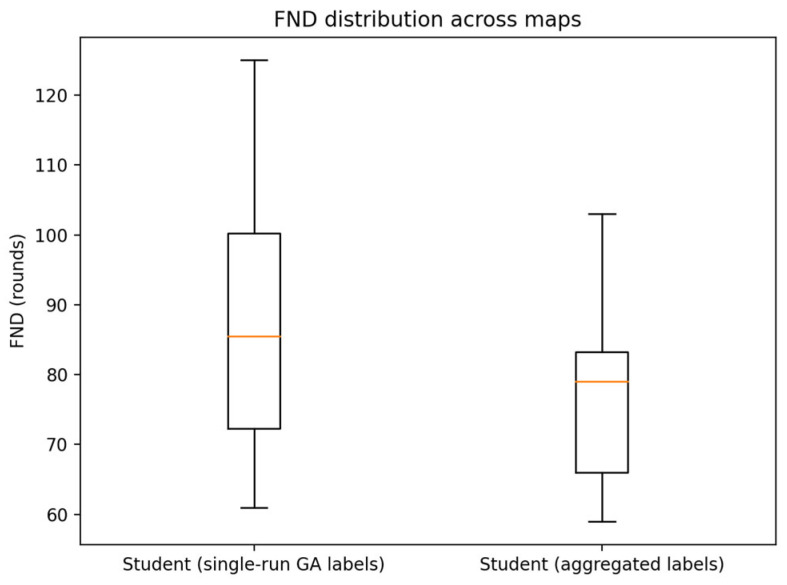
Comparison of FND metrics: Baseline vs. Aggregated-label model.

Evaluation across 10 maps demonstrates that label aggregation yields more stable network lifetime performance. Notably, there was no significant discrepancy in FND metrics between the two models. These findings suggest that the proposed HEOCP framework is robust and not overly sensitive to the pseudo-label noise inherent in the GA process. Figure 17 illustrates the results of our analysis.

## 6. Conclusions

In this study, we introduced the Energy-Optimized Clustering Protocol. This is a new way to cluster WSNs that uses modeling of radio energy consumption and a hybrid optimization framework that combines GA with deep learning through ResNet-50. Our method effectively balances the consumption of energy among sensors by analytically constructing a threshold-based CH selection zone and determining the optimal number of clusters. The hybrid GA–ResNet-50 model makes it possible to select a CH in real time, which means that network operation does not need to utilize expensive heuristic optimization.

Simulation results across several network sizes show that HEOCP extends WSN’s lifetime more effectively compared to traditional methods like LEACH and GA-enhanced clustering. In particular, limiting CH selection to areas with low consumption of energy and dynamically determining how many CHs were needed were both important in postponing the death of the first sensor and making more efficient use of energy overall. The ResNet-50 model also accurately mimicked GA decision-making, which made it possible to cluster data in a way that was both scalable and efficient without losing performance. The results of this study demonstrate the importance of integrating analytical energy models with intelligent learning frameworks to develop more flexible and sustainable WSN protocols. Overall, HEOCP provides both theoretical insights and practical techniques for extending WSN lifetime, and it points to promising directions for future research in energy-aware network design.

Moreover, our setup assumes deployment of homogeneous network nodes in an obstacle-free environment, which allows for problem simplification. However, heterogeneous networks with obstacles may better reflect certain WSN application scenarios, and energy-efficient strategies for such environments represent a promising direction for future research.

## Figures and Tables

**Table 1:** Variables descriptions.

Name	Description
$E_{r}$	Energy use by a wireless sensor receiving *l* bits.
$E_{e}$	Energy used by the electronic circuit to send and receive signals.
$E_{BF}$	The energy consumed by beam forming.
$E_{L}(d)$	Radio transmission of *l* bits messages consumes energy when *d* exceeds a threshold.
$\;\;E_{S}(d)$	Short transmission distances reduce radio transmission energy consumption of *l* bit information.
$\epsilon_{s}$	Energy used by the amplifier to transport information short distances.
$\epsilon_{l}$	Energy used by the amplifier to transport information long distances.
$E_{ar}$	Energy overhead induced by frequent CH selection.
$E_{add}$	When these sensors are CHs, their added radio transmission energy usage.
$d_{r}$	The expected sensor-to-sink distance for CHs over 87.7 m away.
$E_{EVA}$	Expected additional energy consumption by sensors less than *T* from sink.
$f_{d}\left(r\right)$	PDF
$F_{d}\left(r\right)$	CDF
$d_{e}$	Expected distance between sensors omitted from CH selection and sink.
$E_{cs}$	Average energy consumption from all CHs to the sink.
$E_{EVL}$	Expected energy usage for sensors greater than T from the sink transmitting *l* bits of signal.
$d_{ic}$	Expected distance between node and CH.
X	Half the length of the map side.
*M*	The side length of the map.
*n*	The number of sensors.
*k*	The number of CHs.
*T*	The threshold value used to constrain the CH selection range.

**Table 2:** Comparison results of CNN, ResNet 20, and ResNet 101.

Deep Learning Model	Average WSN Operational Rounds
CNN	325.1
ResNet 50	338.3
ResNet 101	261.4

**Table 3:** Comparison of runtime between HEOCP and GA.

	300 m	400 m	500 m
HEOCP	1.96 s	1.98 s	1.96 s
GA	318.73 s	237.48 s	136.41 s

**Table 4:** Parameters used in simulation experiments.

Network Parameters	Values
Network size	300 × 300, 400 × 400, 500 × 500 $m^{2}$
Number of sensors	100, 450, 800, 1250
Packet size	2000 bits
Initial energy	0.5 J/sensor
Transmitter energy	50 nJ/bit
Receiver energy	50 nJ/bit
Amplification energy for short distance, $E_{efs}$	10 pJ/bit/$m^{2}$
Amplification energy for long distance, $E_{efl}$	0.0013 pJ/bit/$m^{4}$
Consumption of energy during beam formation	5 nJ/bit
Maximum number of Iteration	100
Rleft	$ceil\left(\frac{1}{the\;number\;of\;CH}\right)$
Packet length	4000 bits
Initial population size	50
Maximum generations	100
Crossover rate	0.8
Number of offspring per crossover	40
Mutation rate	0.3
Crossover operator	Single Point Crossover
InitialLearnRate	0.01
Max Epochs	80
Batch size	32
Validation Frequency	100

**Table 5:** The Parameters for The Clustering.

Network Size	CH Selection Threshold *T*	Cluster Number *k*
300 × 300 $m^{2}$	165 m	27
400 × 400 $m^{2}$	200 m	19
500 × 500 $m^{2}$	275 m	15

**Table 6:** Descriptive statistics of FND on 300 × 300 maps.

Method	Mean	Std	Var	p5	p10	Median	CV	IQR	95% CI
HEOCP	491.47	50.48	2548.67	413.90	424.90	509.50	0.06	64.50	[468.88, 514.05]
GA	487.90	53.60	2872.54	426.50	431.00	482.00	0.11	66.50	[449.56, 526.24]
GAmobile	365.40	175.30	30731.60	165.60	187.20	355.50	0.48	264.75	[239.99, 490.81]
DCRN	330.20	78.05	6092.40	250.95	273.90	304.50	0.24	85.25	[274.36, 386.04]
GWO	204.30	27.66	764.90	171.75	178.50	196.50	0.14	31.50	[184.52, 224.08]
PSO	155.60	8.87	78.71	141.25	143.50	157.50	0.18	9.00	[140.18, 180.62]
GACHE	160.40	28.26	798.71	133.05	137.10	145.00	0.06	38.50	[149.25, 161.95]
L+GNN	101.00	39.00	1520.89	45.70	48.40	116.00	0.39	54.00	[73.10, 128.90]
CMWSN	47.00	12.00	144.00	27.05	31.10	48.50	0.26	10.00	[38.42, 55.58]

**Table 7:** Descriptive statistics of FND on 400 × 400 maps.

Method	Mean	Std	Var	p5	p10	Median	CV	IQR	95% CI
DCRN	282.30	21.03	442.46	248.70	251.40	285.00	0.30	10.75	[222.28, 344.32]
HEOCP	268.47	57.32	3285.36	196.90	205.20	271.50	0.18	78.00	[233.64, 303.30]
CMWSN	226.10	20.90	436.77	199.30	205.10	224.00	0.07	31.75	[267.25, 297.35]
GA	184.10	55.43	3072.68	178.00	183.40	184.00	0.09	105.00	[211.15, 241.05]
GAmobile	174.33	32.58	1061.50	145.80	145.80	164.00	0.19	63.00	[149.29, 199.38]
PSO	90.30	21.74	472.68	34.35	38.40	98.00	0.10	15.75	[103.86, 120.14]
GACHE	112.00	11.37	129.33	100.00	100.00	111.50	0.19	12.50	[62.30, 81.70]
GWO	72.00	13.56	183.87	52.35	53.70	73.50	0.24	11.25	[44.95, 63.65]
L+GNN	17.70	12.39	153.43	8.05	12.30	13.50	0.70	23.25	[19.82, 59.58]

**Table 8:** Descriptive statistics of FND on 500 × 500 maps.

Method	Mean	Std	Var	p5	p10	Median	CV	IQR	95% CI
CMWSN	269.30	66.62	4438.21	191.00	197.30	264.50	0.25	88.25	[221.64, 316.96]
GA	121.50	39.77	1581.54	74.00	74.70	116.50	0.33	50.75	[93.05, 149.95]
HEOCP	106.03	11.83	139.96	84.00	93.90	108.50	0.08	17.50	[100.02, 112.05]
GAmobile	104.00	40.09	1607.37	65.00	65.80	88.00	0.39	38.25	[75.32, 132.68]
DCRN	42.30	7.54	56.90	31.00	32.70	43.50	0.18	11.25	[36.90, 47.70]
GACHE	25.40	5.82	33.83	18.00	19.60	26.00	0.23	6.50	[21.24, 29.56]
GNN	19.22	11.60	134.49	7.00	11.00	17.00	0.60	7.00	[10.31, 28.13]
GWO	12.30	5.58	31.12	4.00	5.70	14.00	0.45	8.00	[8.31, 16.29]
PSO	7.50	3.63	13.17	3.00	3.90	8.00	0.48	6.00	[8.31, 16.29]

**Table 9:** Quantitative analysis of GA label consistency via Jaccard Index.

States	Mean ± Std	Min, Median, Max
50	0.366 ± 0.025	0.332, 0.363, 0.425

## Data Availability

The dataset used in this study was generated at random. The experimental dataset is available on request from the corresponding author.

## Abbreviations

The following abbreviations are used in this manuscript:

HEOCP	Hybrid energy-optimized clustering protocol
CH	Cluster head
WSN	Wireless sensor networks
GA	Genetic algorithm
LEACH	Low-energy adaptive clustering hierarchy
DCRN	Dynamic clustering-based methodology and frame relay nodes
CMWSN	Clustering mobile wireless sensor networks
GWO	Grey Wolf Optimizer
PSO	Particle swarm optimization
GACHE	Genetic algorithm for cluster head election
L+GNN	LEACH+GNN
GAmobile	LEACH+GA+MobileNet V3-small
FND	First node dead
